# Incorporating quantitative variables into linkage analysis using affected sib pairs

**DOI:** 10.1186/1753-6561-1-s1-s98

**Published:** 2007-12-18

**Authors:** Yen-Feng Chiu, Jeng-Min Chiou, Yi-Shin Chen, Hui-Yi Kao, Fang-Chi Hsu

**Affiliations:** 1 Division of Biostatistics and Bioinformatics, National Health Research Institutes, 35 Keyan Road, Zhunan, Miaoli 350 Taiwan, Republic of China; 2 Institute of Statistical Science, Academia Sinica, 128 Academia Road, Taipei 115, Taiwan, Republic of China; 3 Department of Nursing, Yuanpei University, 306 Yuanpei Street, Hsinchu 30015, Taiwan, Republic of China; 4 Department of Biostatistical Sciences, Wake Forest University School of Medicine, Medical Center Boulevard, Winston-Salem, North Carolina 27157, USA

## Abstract

Rheumatoid arthritis is a complex disease in which environmental factors interact with genetic factors that influence susceptibility. Incorporating information about related quantitative traits or environmental factors into linkage mapping could therefore greatly improve the efficiency and precision of identifying the disease locus. Using a multipoint linkage approach that allows the incorporation of quantitative variables into multipoint linkage mapping based on affected sib pairs, we incorporated data on anti-cyclic citrullinated peptide antibodies, immunoglobulin M rheumatoid factor and age at onset into genome-wide linkage scans. The strongest evidence of linkage was observed on chromosome 6p with a *p*-value of 3.8 × 10^-15 ^for the genetic effect. The trait locus is estimated at approximately 45.51–45.82 cM, with standard errors of the estimates range from 0.82 to 1.26 cM, depending on whether and which quantitative variable is incorporated. The standard error of the estimate of trait locus decreased about 28% to 35% after incorporating the additional information from the quantitative variables. This mapping technique helps to narrow down the regions of interest when searching for a susceptibility locus and to elucidate underlying disease mechanisms.

## Background

Several biomarkers, including anti-cyclic citrullinated peptide (anti-CCP) antibodies and immunoglobulin M rheumatoid factor (IgM RF), are used to characterize rheumatoid arthritis (RA). Anti-CCP antibodies and IgM RF are important surrogate markers for diagnosis and prognosis in RA. The genetic mechanism of these biomarkers might directly underlie disease status. If not, the quantitative trait loci (QTL) might be linked to the loci responsible for RA, or the quantitative trait might interact phenotypically with RA. Hence, incorporating the quantitative trait into analyses will increase our ability to map the genes that predispose to RA [1]. In addition, age at onset is associated with an increased risk of RA [2,3]. Therefore, we also incorporated age of onset into our analysis to increase the power of localizing disease locus.

Recently, Liang et al. [4] proposed a robust multipoint linkage analysis approach using affected sib pairs, which provides an estimate of the genetic effect and the location of the disease locus *τ*, along with sampling uncertainty to help investigators to narrow down chromosomal regions putatively harboring disease locus. The genetic effect is denoted by "*C*", and the value, (1 + *C*)/2, characterizes the probability of an affected sib pair sharing the same allele at *τ *from the parent. Chiou et al. [5] extended this method to estimate *C *nonparametrically by incorporating the information from either a quantitative trait or covariate, aiming to estimate *τ *more efficiently. Hence, in the present study, we incorporate information from several quantitative variables associated with RA, including anti-CCP, IgM RF, and age at onset, into our linkage mapping to enhance the efficiency of identifying the locus responsible for RA.

## Methods

### Materials

Data from a total of 1096 affected sib pairs from 757 multiplex families in the North American Arthritis Consortium study (NARAC) were included. Only 615 or 627 sib pairs (depending on the chromosomal regions) had genotype information, and thus these were used for our analysis. The NARAC multiplex families contain 8017 individuals, about 90.6% of whom are Caucasian, the rest are Hispanic (5.42%), African-American (2.92%), Native American (0.57%), and Asian (0.51%). When we performed the analyses using the subset of Caucasians and the whole data set, the results were quite similar; hence, we report only the results from whole data set here.

A total of 375 microsatellite markers were used in the analyses. The genotype data for 12 affected sib pairs on chromosomes 1 to 11, 13 to 16, and 19 to 22 were missing; therefore, 615 affected sib pairs were available for those chromosomes and 627 affected sib pairs were available for chromosomes 12, 17, and 18. Due to the missingness of the incorporated quantitative variables, the total number of affected sib pairs being included in the analysis varied from 588 to 622, depending on the quantitative variable and on the genotype data on the chromosomal regions.

### Linkage approaches

The GeneHunter program was used to calculate identity by descent (IBD) sharing of affected sib pairs. The GeneFinder program was used to perform the linkage mapping with estimates of *C *and *τ *based on the phenotype of disease status only. It provides estimates of *τ *and its 95% confidence interval as well as the *p*-value of testing whether *C *= 0 (namely, if the linkage is present). A Fortran program developed by Chiou et al. [5] was applied to estimate *τ *and its 95% confidence interval where *C *was a non-linear function of the quantitative variable and was estimated nonparametrically. *C *as a function of a quantitative covariate was estimated by ${\hat{\beta }}_{0}$ such that $\hat{\beta }=({\hat{\beta }}_{0},{\hat{\beta }}_{1})$ was the minimizer of the following kernel weighted least squares function with respect to $\beta =({\hat{\beta }}_{0},{\hat{\beta }}_{1}),\sum_{i=1}^{n}[(S_{i}^{*}(\tilde{\tau })-1)-\beta_{0}-\beta_{1}(g_{i1}-g_{1}){]}^{2}K_{2}(H^{-1}(g_{1}-g_{i1}))$, where $S_{i}^{*}(\tilde{\tau })$ was the imputed IBD sharing at ($\tilde{\tau }$), an estimate of *τ*. *g*_*i*1 _= *g*_1_(*x*_*i*1_, *x*_*i*2_) = (*x*_*i*1 _+ *x*_*i*2_)/2, (*x*_*i*1_, *x*_*i*2_) were the values of the quantitative variable for the *i*^th ^affected sib pair. *K*_2 _was a kernel function, and *H *was a non-singular square bandwidth matrix.

Because this method is an extension to the approach proposed by Liang et al. [4], it is also a robust approach in that no assumption about the genetic mechanism is required other than that the region contains no more than one susceptibility locus for the qualitative trait. No assumption about the underlying genetic mechanism of an incorporated quantitative trait is required.

We compared the results from incorporating the quantitative variable with those obtained from the GeneFinder search to evaluate the efficiency gained by the additional information from a given quantitative variable.

## Results

**Figure 1:**
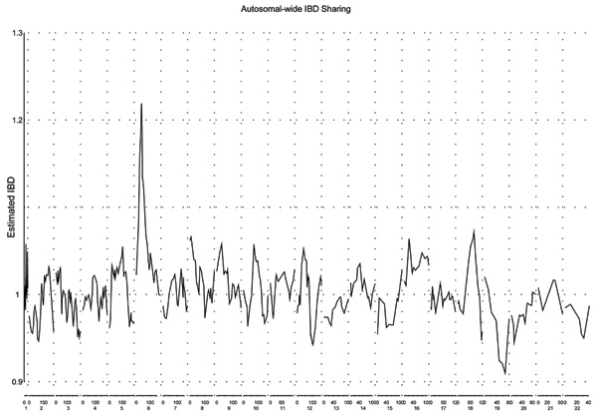
Autosomal-wide estimated IBD sharing for affected sib pairs.

We plotted the average estimated IBD sharing from affected sib pairs along the autosomal chromosome regions, as demonstrated in Figure 1. The peak on chromosome 6 was distinguishable from the others. The GeneFinder search results showed that the disease locus (*τ*) was estimated to be at 45.87 cM, with a 95% confidence interval of [43.39, 48.34] on chromosome 6 (Table 1). C is estimated to be 0.22 with a standard error of 0.028 (*p*-value = 3.80 × 10^-15^). Hence, the probability of an affected sib pair sharing the same allele from a parent is estimated to be 0.61 at *τ*. Other regions showing evidence of linkage included the region at around 91.6 cM on chromosome 5 (*p *= 0.04), 7.09 cM on chromosome 8 (*p *= 0.0027), 40.11 cM on chromosome 9 (*p *= 0.02), 84.21 cM on chromosome 10 (*p *= 0.047), 49.83 cM on chromosome 16, and 70.22 cM on chromosome 18 (*p *= 0.043) (Table 1).

**Table 1:** Autosomal-wide linkage mapping without incorporating a quantitative variable

Chromosome	$\hat{\tau }$ (SE)	95% C.I. for *τ*	$\hat{C}$ (SE)	*p*-value for testing *H*_0_:*C *= 0
1	142.81 (13.6)	[116.16, 169.46]	0.04 (0.031)	0.095
2	207.79 (12.39)	[183.52, 232.07]	0.032 (0.03)	0.14
3	24.24 (14.03)	[-3.26, 51.74]	0.023 (0.029)	0.21
4	178.47 (53.35)	[73.91, 283.02]	-0.006 (0.03)	1
5	91.6 (8.49)	[74.96, 108.24]	0.063 (0.036)	0.04
6	45.87 (1.26)	[43.39, 48.34]	0.219 (0.028)	3.80 × 10^-15^
7	90.37 (36.11)	[19.59, 161.14]	0.014 (0.036)	0.35
8	7.09 (4.78)	[-2.28, 16.46]	0.08 (0.029)	0.0027
9	40.11 (6.82)	[26.74, 53.48]	0.067 (0.033)	0.02
10	84.21 (8.01)	[68.51, 99.91]	0.049 (0.029)	0.047
11	67.67 (25.9)	[16.9, 118.43]	0.034 (0.045)	0.22
12	52.16 (13.49)	[25.72, 78.59]	0.036 (0.032)	0.13
13	90.07 (17.77)	[55.24, 124.9]	-0.022 (0.028)	1
14	49.75 (15.29)	[19.78, 79.71]	0.032 (0.037)	0.19
15	60.55 (6.24)	[48.31, 72.78]	-0.042 (0.027)	1
16	49.83 (6.90)	[36.3, 63.36]	0.064 (0.035)	0.034
17	23.39 (36.58)	[-48.31, 95.09]	-0.01 (0.027)	1
18	70.22 (8.80)	[52.97, 87.47]	0.056 (0.033)	0.043
19	82.32 (4.43)	[73.63, 91.01]	-0.098 (0.027)	1
20	18.75 (10.66)	[-2.15, 39.64]	-0.059 (0.038)	1
21	Not convergent			
22	34.74 (4.94)	[25.06, 44.42]	-0.043 (0.027)	1

By incorporating anti-CCP into the linkage mapping (Table 2), *τ *was estimated to be 45.51 cM on chromosome 6. The standard error decreased from 1.26 to 0.82; thus, the corresponding 95% confidence interval decreased by 1.73 cM. Similarly, by incorporating IgM RF (Table 3) or age at onset (Table 4), the estimates of the disease locus were located at the same region. The corresponding 95% confidence intervals decreased 1.39 cM for RF IgM and 1.59 cM for age at onset. In addition, after incorporating anti-CCP, IgM RF, or age at onset, the standard errors for the estimates of *τ *reduced by roughly 34 to 77% on chromosomes 9, 10, and 18, respectively. The numbers of affected sib pairs with anti-CCP, IgM RF, and age at onset available were 588, 611, and 605, respectively, on chromosomes 1 to 11, 13 to 16, and 19 to 22; and were 600, 622, and 617, respectively, on chromosomes 12, 17, and 18, less than the numbers of affected sib pairs of 615 and 627 on these two sets of chromosomal regions, respectively, due to the presence of missing quantitative variables. The reduction of standard errors for the estimates of *τ *when incorporating these quantitative variables were in fact computed based on fewer sib pairs.

**Table 2:** Autosomal-wide linkage mapping incorporating anti-cyclic citrullinated peptide antibodies

Chromosome	$\hat{\tau }$ (SE)	95% C.I. for *τ*
1	209.99 (5.86)	[198.5,221.48]
2	211.87 (3.74)	[204.54,219.2]
3	21.28 (2.31)	[16.75,25.81]
4	59.68 (2.92)	[53.957,65.4]
5	104.74 (2.99)	[98.88,110.6]
6	45.51 (0.82)	[43.9,47.12]
7	23.55 (5.07)	[13.61,33.487]
8	11.6 (4.24)	[3.29,19.91]
9	44.93 (4.53)	[36.05,53.81]
10	86.64 (5.12)	[76.6,96.68]
11	129.23 (4.44)	[120.53,137.93]
12	21.98 (3.07)	[15.96,28]
13	82.53 (1.83)	[78.943,86.12]
14	44.547 (5.91)	[32.96,56.13]
15	55.08 (4.025)	[47.19,62.97]
16	102.13 (6.27)	[89.84,114.42]
17	56.08 (3.37)	[49.47,62.685]
18	69.67 (4.02)	[61.79,77.55]
19	83.68 (3.57)	[76.68,90.68]
20	52.47 (4.97)	[42.73,62.211]
21	Not convergent	
22	37.03 (3.76)	[29.66,44.4]

**Table 3:** Autosomal-wide linkage mapping incorporating rheumatoid factor-immunoglobulin M

Chromosome	$\hat{\tau }$ (SE)	95% C.I. for *τ*
1	110.44 (2.052)	[106.42, 114.46]
2	200.55 (3.40)	[193.89, 207.21]
3	122.41 (3.46)	[115.628, 129.19]
4	143.66 (3.43)	[136.9372, 150.38]
5	82.21 (7.95)	[66.64, 97.79]
6	45.57 (0.91)	[43.79, 47.35]
7	32.2 (3.42)	[25.497, 38.9]
8	9.55 (4.54)	[0.652, 18.45]
9	44.04 (4.028)	[36.15, 51.93]
10	91.85 (4.46)	[83.1, 100.6]
11	129.97 (5.69)	[118.82, 141.122]
12	56.33 (3.98)	[48.53, 64.13]
13	33.75 (3.48)	[26.965, 40.57]
14	34.88 (3.45)	[28.12, 41.64]
15	57.6 (4.10)	[49.56, 65.64]
16	58.88 (6.26)	[46.61, 71.14]
17	115.57 (6.062)	[103.689, 127.45]
18	72.04 (4.41)	[63.4, 80.68]
19	84.4 (3.44)	[77.66, 91.14]
20	55.29 (3.37)	[48.68, 61.9]
21	Not convergent	
22	27.36 (8.94)	[9.84, 44.88]

**Table 4:** Autosomal-wide linkage mapping incorporating age at onset of rheumatoid arthritis

Chromosome	$\hat{\tau }$ (SE)	95% C.I. for *τ*
1	238.72 (3.41)	[235.1, 242.34]
2	145.72 (2.43)	[140.96, 150.48]
3	59.623 (3.89)	[55.76,63.49]
4	168.66 (5.999)	[156.9, 180.42]
5	73.68 (11.10)	[51.92, 95.44]
6	45.82 (0.86)	[44.14, 47.5]
7	31.46 (4.36)	[22.92, 40]
8	10.06 (4.76)	[0.739, 19.38]
9	42.92 (4.13)	[34.83, 51.02]
10	80.51 (2.79)	[75.04, 85.98]
11	103.53 (3.52)	[96.63, 110.43]
12	10.35 (3.38)	[3.73, 16.98]
13	79.09 (2.67)	[73.86, 84.32]
14	54.64 (5.038)	[44.77, 64.514]
15	59.98 (6.31)	[47.61, 72.348]
16	44.73 (4.45)	[36.01, 53.45]
17	22.47 (4.22)	[14.21, 30.74]
18	79.53 (2.024)	[75.56, 83.5]
19	79.46 (3.29)	[73, 85.92]
20	83.7 (2.13)	[79.53, 87.87]
21	39.6 (5.61)	[28.6, 50.6]
22	34.15 (3.23)	[27.82, 40.48]

## Conclusion

The diagnosis of RA is generally based on the presence and titer of specific autoantibodies (IgM RF and anti-CCP); joint involvement, according to the joint alignment and motion (JAM) score; the presence and extent of erosive disease on hand/wrist radiographs; functional status according to the health assessment questionnaires (HAQ) scores, age and calendar year of RA onset; the presence of nodules or other extra-articular manifestations; and the presence of other autoimmune diseases [3]. Hence, searching for the disease susceptibility loci based on the disease status defined by a threshold process might miss out a lot of information contained in the quantitative variables.

We demonstrated that the efficiency of the disease locus localization was greatly improved by the incorporation of quantitative variables related to RA. By applying this approach, the investigators would be able to narrow down the regions of interest when searching for disease susceptibility loci. The significance of the improvement in the location estimate could be assessed by a bootstrap method. We are currently conducting systematic simulation studies to assess the improvement, the results will be reported elsewhere.

The estimated standard error of $\hat{\tau }$ after incorporating anti-CCP (0.82) was smaller than that after incorporating IgM RF (0.91), suggesting that anti-CCP provides slightly more information about RA than IgM RF, consistent with the results from other studies [3]. The estimated standard error of $\hat{\tau }$ after incorporating age at onset was 0.86, similar to what was observed for anti-CCP, indicating that age at onset contains as much information about RA as anti-CCP and is also related to the underlying genetic mechanism of RA. The confidence regions on all the chromosomes with linkage evidence were narrowed down by incorporating one of the three quantitative covariates. Among them, the confidence region on chromosomes 5 had the greatest reduction by incorporating anti-CCP, while incorporating IgM RF or age at onset had the largest effect on shortening the confidence region on chromosome 18. For a specific chromosomal region, the reduction of the standard error for the trait locus estimate varied by the quantitative covariate incorporated, indicating genetic heterogeneity existed among these RA-related quantitative variables. By examining the efficiency gain through incorporating a quantitative covariate, we are not only able to locate the susceptibility locus more precisely but also to identify genes related to distinct pathways associated with different quantitative variables. These findings suggested that incorporations of quantitative RA phenotypes or RA-related covariates did increase the power of the identification of genes in this approach and therefore help elucidate underlying disease mechanisms of RA.

## Competing interests

The author(s) declare that they have no competing interests.
